# Experimental study on mechanical properties and microstructure of cement-based expansion grouting materials for broken coal mass

**DOI:** 10.1371/journal.pone.0328492

**Published:** 2025-08-18

**Authors:** Feng Zhang, Jinxiao Liu, Jiawei He, Kai Zhang, Zhongcheng Qin

**Affiliations:** 1 School of Energy and Mining Engineering, Shandong University of Science and Technology, Qingdao, China; 2 National Demonstration Center for Experimental Mining Engineering Education, Shandong University of Science and Technology, Qingdao, China; Shandong University of Technology, CHINA

## Abstract

As the excavation depth of the tunnel increases, the stress on the surrounding rock increases, and the tunnel becomes fragmented, especially in coal tunnels, which require grouting reinforcement. Conventional materials are inadequate for the grouting and reinforcing demands of this type of surrounding rock; therefore, it is essential to create grouting materials with specific expansion properties. First, the orthogonal test method was employed to optimize the expansion grouting materials ratio. Compared with traditional grouting materials, it exhibits certain expansibility along with good fluidity and strength. Based on a comprehensive evaluation of the mechanical properties of the material, the optimal formulation was determined as follows: 10% UEA expansive agent, 5% early-strength agent, and 1.2% water-reducing agent. Additionally, Through SEM tests and grouting reinforcement test of broken coal or rock, it was found that expansive grouting materials can effectively fill micro-fractures in coal rock, enhance the integrity of the surrounding rock, and improve its mechanical properties. The findings indicate that the recovery strength of coal and rock using expansion grouting materials at 7 days is 0.18 and 0.1 greater than that of pure cement; at 28 days, the recovery strength of coal and rock is 0.27 and 0.15 higher than that of pure cement. Ultimately, the expansion grouting materials is employed in engineering applications. Following the application of anchor grouting and expansion grouting materials, the maximum deformation of the roof at the roadway intersection measures 125 mm, while the maximum deformation on both sides reaches 190 mm. The distortion of the surrounding rock of the roadway is well managed. This is essential for the reinforcement of tunnels within shattered surrounding rock and for the secure extraction of coal in mines.

## 1 Introduction

The superficial coal reserves in China are progressively depleting, leading to deeper coal mining operations. The safety of coal mine roadways post-excavation is intimately linked to the fragmentation of surrounding rock as mining depth increases. The greater the fragmentation of the surrounding rock, the diminished safety of the roadway, and the increased support costs. In light of the support of coal mine fractured surrounding rock roadways [1–3], scholars have conducted extensive scientific investigations. Theories and technologies such as loose ring theory, joint support theory [4–6], U-type steel shrinkage support, and anchor cable networks [7–11] have been progressively developed. The aforementioned has significantly influenced the safety of coal mine roadways. However, when the surrounding rock of the roadway is relatively loose or fractured, conventional support methods cannot establish a solid bearing system. Consequently, it is infeasible to avert the emergence of roadway support failures, bottom drum issues, roof subsidence, or other phenomena, which significantly contribute to roadway collapse safety incidents, adversely impacting coal mine productivity. For this roadway characterized by fractured surrounding rock, therapy is typically administered using Grouting consolidates the fractured surrounding rock [12–17], enhancing its structural integrity and physical-mechanical properties, thereby augmenting its bearing capacity and establishing a dependable foundation for the anchor rod [17–26].

Researchers employed scanning electron microscopy (SEM) to examine the physical properties of grouting materials through microscopic testing and performance analysis [27–31]. Typically, there is no substantial distinction between the thermal analysis and infrared spectrum analysis of expanded and unexpanded materials. Nevertheless, the micromorphology of the hydration observed via scanning electron microscopy is markedly distinct [32–36]. The hardening process of conventional Materials exhibits a specific degree of dry shrinkage, leading to a low density in the traditional anchor injection surrounding rock fissures. This results in a limited enhance Materials of the overall strength of the roadway surrounding rock, making it challenging to establish an effective structure. This study proposes the use of grouting material with specific expansion properties [37–42] to reinforce the extensive area of fractured roadway, thereby creating an effective and construction.

Due to the low strength and poor fluidity of traditional expansive grouting materials, this study utilized cement, expansion agent, early-strength agent, and water-reducing agent as raw materials to prepare a grouting material with certain expansive properties and adequate strength, employing the orthogonal test method [42–47]. The mechanical properties of the grouting solid were evaluated, and the microstructure was examined and assessed using SEM. Employ the intersection at the inlet rock gate of -808m phase and the track tunnel of 1304 face as a case study for the actual implementation of the innovative expansion grouting materials. The monitoring results indicate that the grouting material with certain expansion properties may successfully regulate the deformation of the surrounding rock. This is essential for the reinforcement of tunnels within shattered surrounding rock and for the secure extraction of coal in mines.

## 2 Materials and methods

### 2.1 Expansion grouting materials and experimental design

**Fig 1:**
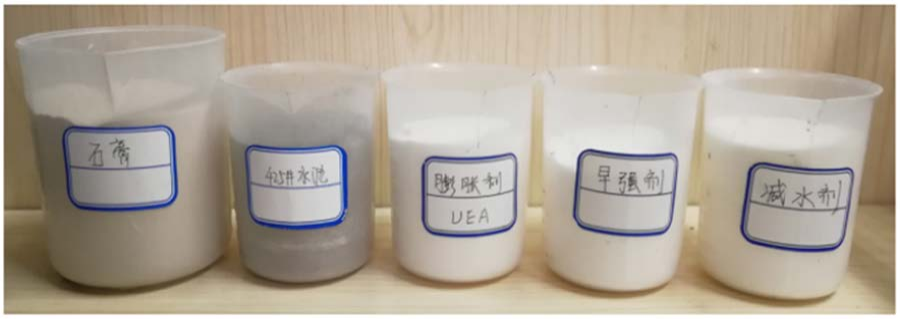
Sample raw materials.

To enhance the strength and fluidity of the grouting material while expansion properties, water-reducing agent and early-strength agent were added to the 425# Portland cement, expansive agent and gypsum. Fig 1 illustrates the raw materials utilized in the ratio optimization test of the novel expansion grouting materials. It comprises 425# Portland cement, UEA expansion agent, chloride early strength agent, polycarboxylate superplasticizer and gypsum. Table 1 displays the physical attributes of each substance.

**Table 1:** Physical properties of grouting raw materials.

Name		Component	Origin	Density (g/m^3^)	Dosage (%)	Compressive strength (MPa)	Initial set (min)	Final set (min)
Material	Gypsum	CaSO_4_·1/2H_2_O	Yantai, Shandong	2.3		7.5-9.0	5 ~ 8	12 ~ 16
	Cement of 425#	3CaO·SiO_2_ 2CaO·SiO_2_	Jialingguan, Gansu	3.05-3.15		≥42.5	≥45	<600
Additive	UEA Expanding Agent	C_3_A·3CaSO_4_·32H_2_O	Zhengzhou, Henan	2.88	5 ~ 15	≥42.5	164	558
	Chlorine-salt early Strengthening agent	CaCl_2_	Zhengzhou, Henan	2.65g/cm^3^	3 ~ 5		+60	+90
	Polycarboxylate superplasticizer	Grafting polycopolymers of carboxylic acids	Chengdu, Sichuan	1.025	0.4 ~ 1.2	+59	+15	+10

According to relevant literature and the relevant instructions for the use of additives [48–51]. Utilize the orthogonal experimental method to conduct a three-factor, three-level L9 (3^3) orthogonal experimental design. The arrangements of the orthogonal test protocol are presented in Table 2. The proportions of the expanding agent are 5%, 10%, and 15%. The dosages of the water-reducing agent are 3%, 4%, and 5%. Early strength agent dosages are 0.4%, 0.8%, and 1.2%.

**Table 2:** L9(33) orthogonal test scheme arrangement.

Groups	A Expansive agent dosage/%	B Early strength agent dosage/%	C Water reducing agent dosage/%
1	5	3	0.4
2	5	4	0.8
3	5	5	1.2
4	10	3	0.8
5	10	4	1.2
6	10	5	0.4
7	15	3	1.2
8	15	4	0.4
9	15	5	0.8

### 2.2 Determination of physical properties of expansion grouting materials

The determination experiments include the fluidity, viscosity, setting time, water bled rate, expansion ratio, expansion force and the uniaxial compressive strength of the induration.

**Fig 2:**
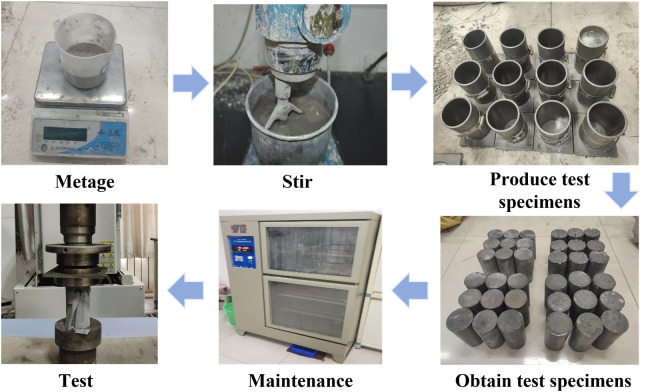
Compressive strength test.

Compressive strength test: As illustrated in Fig 2. ① By introducing the prepared slurry of the expansion grouting materials into the self-designed cylindrical specimen mold. ② Remove the liquid from the mold surface with a scraper and place the cover. ③ Position in an indoor setting for one day and subsequently demold. ④Subsequent to mold release, the specimen shall be labeled and placed in the standard curing chamber maintained at a temperature of 20 ± 2°C and a relative humidity exceeding 95% for a duration of 7 or 28 days. ⑤ Evaluate the compressive strength utilizing the Shimadzu apparatus.

Measurement of expansion rate: ①Smear oil into the expansion rate tester. ② Introduce the slurry of the prepared expansion grouting materials into the container, ensuring to eliminate any bubbles within the slurry. ③ Once the slurry is introduced into the mold, position the glass plate on the top of the mold and place the measuring head at the middle of the glass plate. ④ Calibrate the micrometer to zero and attach the pointer head to the measuring head to evaluate the material’s expansion rate over a duration of 7 days.

**Fig 3:**
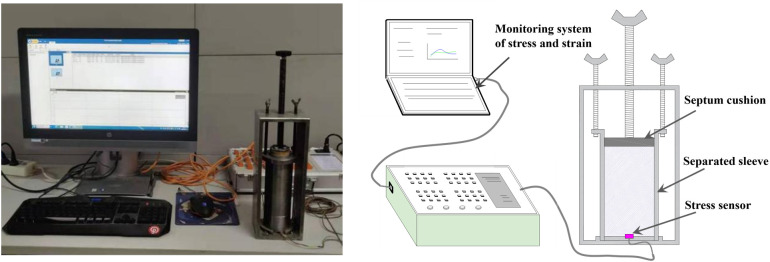
Principle of expansion force test.

Expansion force assessment: ① As illustrated in Fig 3. initially apply oil to the inside wall of the container and the surface of the sealing cover. ② Encase the strain gauge in plastic wrap and position it within the groove at bottom. ③ Subsequently, secure the container firmly in the base slot and affix the container. ④ Finally, Place the slurry into the cavity and place the sealing lid to restrict any volumetric alterations. The vertical expansion force will progressively manifest during the slurry hardening process. The expansion force of the grouting material can be ascertained by monitoring the stress variation in the sensor.

### 2.3 Grouting reinforcement test of broken coal or rock mass

**Fig 4:**
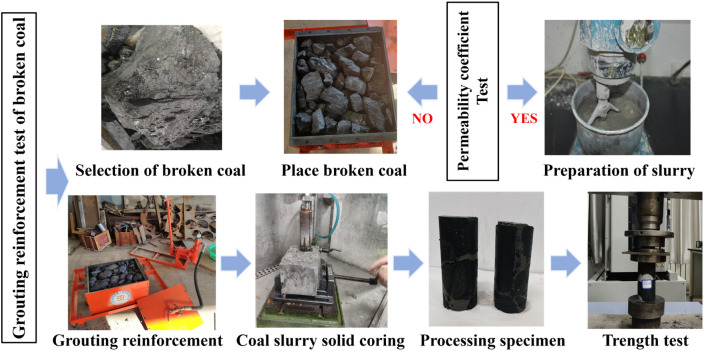
Flow chart of grouting reinforcement test for broken coal mass.

Combined with the actual situation, a simulated grouting device was designed to simulate the grouting reinforcement test of broken coal-rock mass,the test process of grouting reinforcement of broken coal is shown in Fig 4.

**Fig 5:**
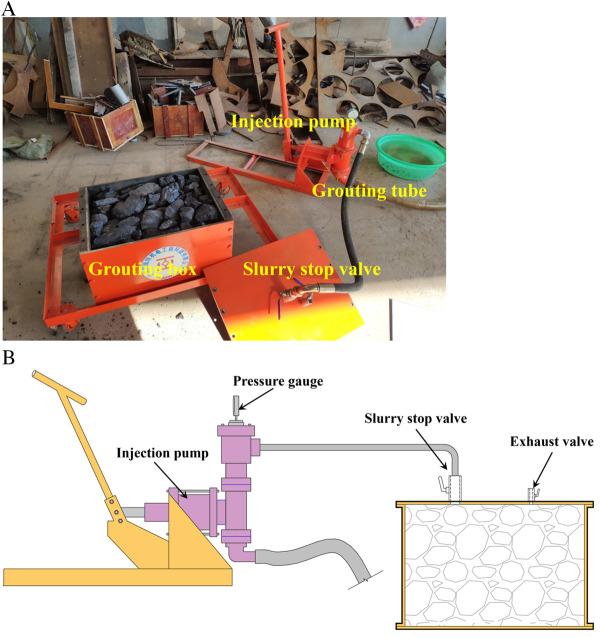
Grouting simulation device. (a) Object image (b) Diagrammatic drawing.

The simulated grouting apparatus is designed to conduct assessments for reinforcement through simulated grouting on fractured coal. The grouting apparatus is mainly composed of injection pump, grouting pipe, Slurry stop valve and grouting box. Fig 5 displays the schematic and physical diagrams of the entire test apparatus. The grouting pump is manual, and its maximum grouting pressure is 6MPa. The size of the grouting box is 500 mm × 400 mm × 300 mm. Its top is equipped with a feed port and an exhaust vent, so that the air in the box can be discharged.

Grouting and mechanical properties test: ① The Shimadzu test machine first measured the whole coal piece’s compressive strength. ② Find the quality of cracked coal or rock specimens. ③ To further create standard specimens, 7d and 28d grouting solids are prepared using the previously mentioned simulated grouting apparatus. ④ Use the Shimadzu tester to find the grouting specimen’s strength. ⑤ Calculate the degree of strength recovery of the broken coal following and grouting reinforcement.

### 2.4 Microscopic tests on expansion grouting materials and grouting solids

**Fig 6:**
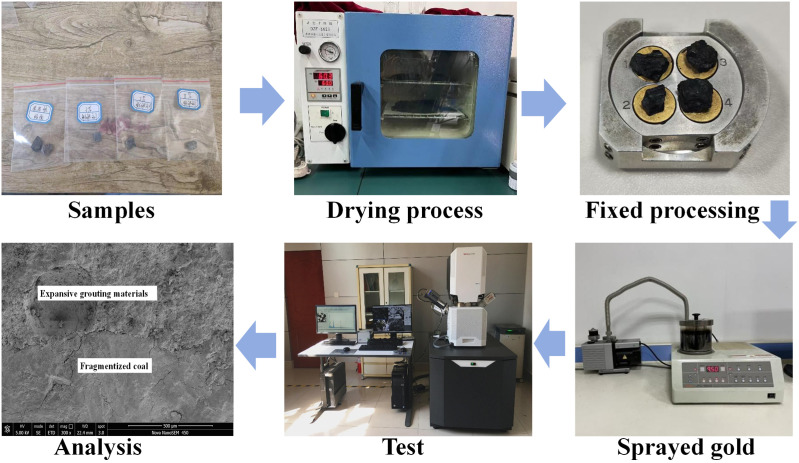
Scanning electron microscope test.

The solidified body developed by varying grouting material concentrations at 28d of age was seen using a scanning electron microscope. Additionally observed was the grouting coal body’s cementing surface. Microscopic analysis of the effect of expansion agent on the structure and characteristics of grouting material follows then. The paper clarifies and analyzes the link between the physical and mechanical change law and its microstructure by relating the microstructural change with the physical and mechanical properties. Then one can gain the internal mechanism of the new expansion grouting substance for reinforcing the crushed coal. The procedure for scanning electron microscope test is illustrated in Fig 6.

## 3 Results and analysis

### 3.1 Physical characteristics and analysis of grouting material

This physical properties of expansion grouting materials tests aims to obtain the physical properties of grouting materials under different ratios through physical property tests. The specific method is to select three samples for each ratio in each group of tests, conduct tests on them, and use the average value of the test results of the three samples as the final physical property data for that ratio to improve the accuracy and representativeness of the results. Assuming there is no interaction between the factors, the results of orthogonal test are shown in Table 3.

**Table 3:** Results of orthogonal test.

Groups	A Expansive agent dosage%	B Early strength agent dosage/%	C Water reducing agent dosage/%	Fluidity (cm)	Initial set(min)	Expansion ratio (%)	Expansion force (MPa)	7-d Compression strength (MPa)	28-d Compression strength (MPa)
1	5	3	0.4	13.5	413	0.092	0.86	26.2	49.6
2	5	4	0.8	16.8	393	0.081	0.69	29.1	50.3
3	5	5	1.2	20.5	361	0.053	0.73	33.5	51.8
4	10	3	0.8	19.7	405	0.101	1.18	25.9	53.2
5	10	4	1.2	20.8	384	0.094	1.09	29.3	55.1
6	10	5	0.4	15.6	376	0.112	1.02	32.6	52.2
7	15	3	1.2	15.5	401	0.102	1.38	26.3	53.8
8	15	4	0.4	14.1	395	0.173	1.21	27.8	52.9
9	15	5	0.8	14.7	365	0.165	1.28	31.2	50.3
Pure cement				13	451	–	5	24.8	28.8

**Fig 7:**
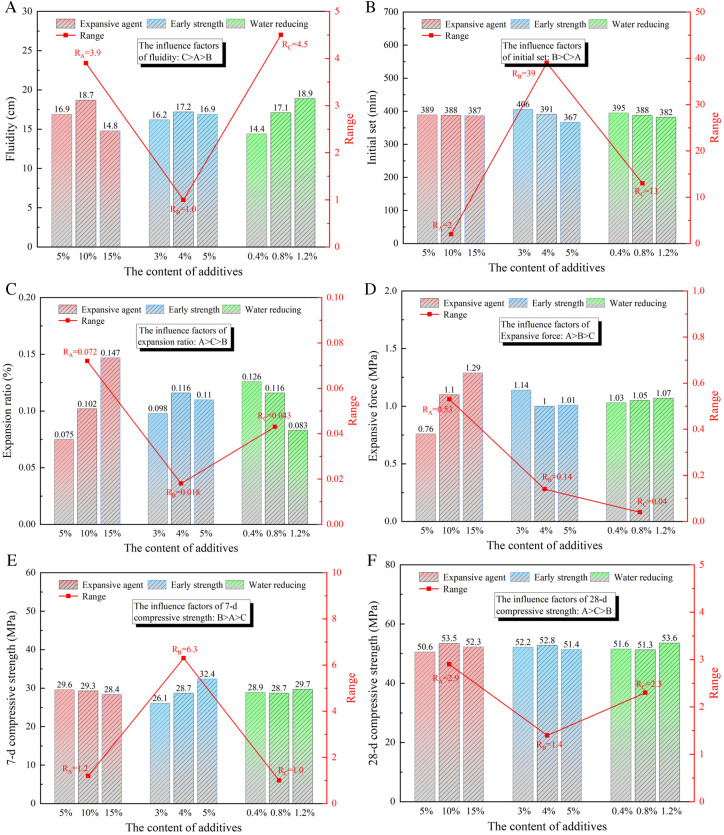
Range analysis of orthogonal test. (a) Fluidity (b) Initial setting time (c) Expansion rate (d) Expansion Force (e) 7-day compressive strength (f) 28-day compressive strength.

Statistical analysis and calculations were conducted on the experimental results of each factor and level. The range analysis method was employed to investigate the influence of multiple parameters on the slurry properties, clarifying the specific effects of different factors on the physical characteristics of the slurry. The statistical data of material physical properties under different ratio and the range analysis results are shown in Fig 7.

(1) Fluidity: From Table 3 and Fig 7a, the fluidity of pure cement is 13 cm. With the increase of water reducing agent, the fluidity of expansion grouting materials increases gradually. The fluidity first increases and then decreases with the increase of the expansion agent incorporation. However, the fluidity changed less with the increase of early strength agent. According to the range analysis, the effect of the water agent is the most obvious, followed by the expansion agent and the early strong agent. The maximum fluidity combination of the expansion grouting materials is 10% UEA expansive agent, 4% early-strength agent, and 1.2% water-reducing agent.
(2) Initial setting time: Table 3 and Fig 7b indicate that the initial setting time of pure cement is 451 minutes. The introduction of early strength agents progressively reduces the initial setting time of the expansion grouting materials. As the quantities of the expansion agent and water-reducing agent rose. The extreme range analysis indicates that the quantity of early strength agent most significantly influences the initial setting time, followed by the water reducer, and lastly the expansion agent. The requisite combination for permitting the expansion grouting materials to attain the minimum initial setting time is 5% UEA expansive agent, 3% early-strength agent, and 0.4% water-reducing agent..
(3) Expansion rate: As indicated in Table 3 and Fig 7c, the expansion grouting materials can entirely counteract the shrinkage of pure cement while also generating additional expansion during the hardening phase. The expansion rate rises with an increase in the expansion agent, albeit at a diminished rate. Conversely, the expansion rate decreases with an increase in the water agent. The extreme range analysis indicates that the quantity of expansion agent most significantly influences the expansion rate, followed by the quantity of water-reducing agent and early strength agent. The optimal combination for the maximum expansion rate of the grouting material is 15% UEA expansive agent, 4% early-strength agent, and 0.4% water-reducing agent.
(4) Expansion Force: According to Table 3 and Fig 7d, the expansion grouting materials generates a specific expansion force upon hardening. The expansion force escalates with the augmentation of the expansion agent above 10%. As the early mixture increases, the swelling force diminishes and subsequently stabilizes; a 4% increase does not impact the swelling force. As the quantity of water-reducing agent escalates. The extreme range analysis indicates that the quantity of expansion agent exerts the most significant impact on the expansion force of the slurry, followed by the quantity of early strength agent, and lastly, the quantity of water reduction agent. The optimal combination of expansion force for the grouting material is 15% UEA expansive agent, 3% early-strength agent, and 1.2% water-reducing agent.
(5) 7-day compressive strength: The 7-day compressive strength of pure cement is 24.8 MPa, as seen in Table 3 and Fig 7e. As the quantity of the early strength agent increases, the 7-day compressive strength of the expansion grouting materials consolidation body progressively rises. The assimilation of expansion agents initially increases and subsequently diminishes. Exceeding a specific threshold of expansion agent will diminish the strength of the grouting material’s consolidated body. The strength of the coal body exhibited minimal variation with the increment of the water-reducing agent. The investigation revealed that the quantity of early strength agent influenced the initial intensity, succeeded by the expansion agent, and subsequently, the quantity of water-reducing agent. The optimal compressive strength combination of the expansion grouting materials at 7 days is 5% UEA expansive agent, 5% early-strength agent, and 1.2% water-reducing agent.
(6) 28-day compressive strength: The 28-day compressive strength of pure cement, as indicated in Table 3 and Fig 7f, is 28.8 MPa. As the quantity of expansion agent increases, the compressive strength of the expansion grouting materials initially rises and thereafter declines. Exceeding a specific threshold of expansion agent results in the formation of many microcracks, thus diminishing compressive strength. As the concentration of water reducer increases, the strength initially diminishes before subsequently rising. The intensity fluctuates minimally with the augmentation of early strength agent integration. The extreme analysis indicates that the quantity of expansion agent influences the strength of the slurry, succeeded by the water-reducing agent, and ultimately the early strength agent. The optimal compressive strength combination of the expansion grouting materials at 28 days is 10% UEA expansive agent, 4% early-strength agent, and 1.2% water-reducing agent.

Based on the range analysis, the primary and secondary order of influencing factors for the six indicators and the most effective level combination were obtained. Comprehensively considering the fluidity, expansion rate, expansion force, and compressive strength to achieve the optimal mix ratio, the optimal mix ratio is 10% expansive agent, 5% early-strength agent, 1.2% water-reducing agent, and a water-cement ratio of 0.6.

### 3.2 Results and analysis of grouting reinforcement of broken coal-rock mass

**Fig 8:**
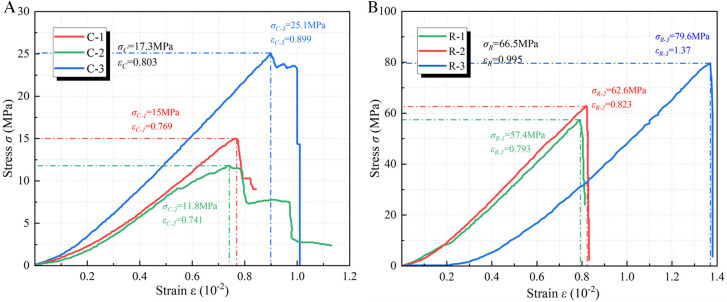
Stress-strain curve of coal-rock specimen. (a) Coal specimen (b) Rock specimen.

Fig 8 illustrates the stress-strain curve of the coal or rock mass specimen. The compressive strength of coal is 17.3MPa, while that of fine sandstone is 66.5MPa.

**Fig 9:**
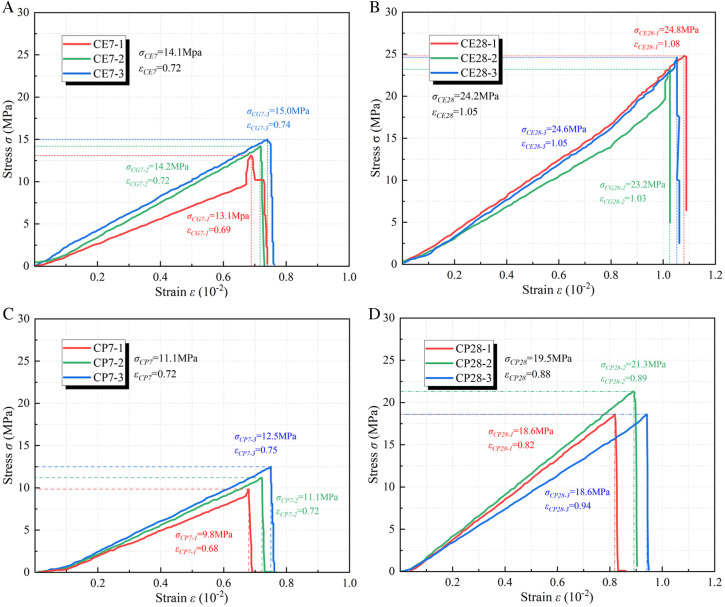
The stress-strain curve of the specimen after grouting reinforcement of broken coal mass. (a) 7-d with expansion grouting materials (b) 28-d with expansion grouting materials (c) 7-d with pure cement (d) 28-d with pure cement.

Fig 9 illustrates the stress-strain curve of the specimen subsequent to the grouting of the fractured coal mass. The compressive strength of the expansion grouting materials after 7 days is 14.1MPa, which is 27% greater than that of pure cement at 11.1MPa. The strength recovery coefficient of expansion grouting materials on coal is 0.82, whereas that of pure cement is 0.64. The compressive strength of the expansion grouting materials after 28 days is 24.2MPa, which is 24% greater than that of pure cement at 19.5MPa. The strength recovery coefficient of expansion grouting materials on coal is 1.40, whereas that of pure cement is 1.13. The reinforcing of expansion grouting materials surpasses that of plain cement and exhibits a favorable recovery coefficient.

**Fig 10:**
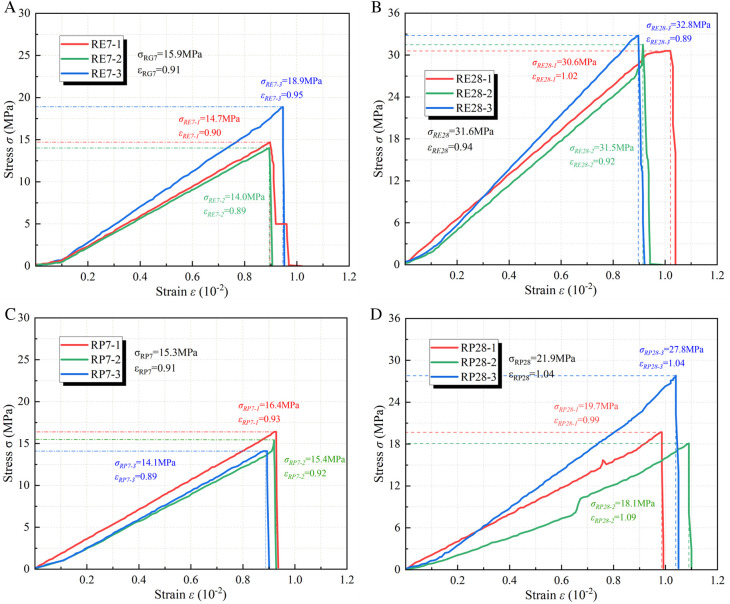
The stress-strain curve of the specimen after grouting reinforcement of broken rock mass. (a) 7-d with expansion grouting materials (b) 28-d with expansion grouting materials (c) 7-d with pure cement (d) 28-d with pure cement.

Fig 10 illustrates the stress-strain curve of the specimen subsequent to the grouting of the fractured rock mass. The compressive strength of the expansion grouting materials, following the grouting reinforcement of the fractured rock mass for 7 days, is 15.9MPa, representing a 4% increase compared to the pure cement strength of 15.3MPa. The strength recovery coefficient of the expansion grouting materials to the rock mass is 0.24, whereas that of pure cement is 0.23. The compressive strength of the expansion grouting materials, after reinforcing the fractured rock mass for 28 days, is 31.6MPa, representing a 44% increase compared to the pure cement strength of 21.9MPa. The strength recovery coefficient of the expansion grouting materials to the rock mass is 0.48, while that of pure cement is 0.33. The strengthening of fractured rock mass using expansion grouting materials resembles that of pure cement in the initial phase, although it surpasses pure cement in subsequent stages.

### 3.3 Microstructure observation and analysis

Portland cement 425# is expanded with a UEA agent. The source of expansion is the development and water absorption of calcium alum stone AFt. The hydration of the UEA expansion agent generates substantial quantities of AFt. Upon the incorporation of the UEA expansion agent into the cement, it will persist in reacting with calcium hydroxide (Ca(OH)_2_), the hydration byproduct of the cement, to generate additional AFt. It intertwines with the C-S-H gel, the hydration product of cement, which subsequently induces a sTable volumetric expansion during the cement hardening process.


$$3CaO\cdot 3{Al}_{2}O_{3}\cdot {CaSO}_{4}\;+\;2Ca{SO}_{4}\;+\;34H_{2}O\to 3CaO\cdot {Al}_{2}O_{3}\cdot 3Ca{SO}_{4}\cdot 32H_{2}O+2{Al}_{2}O_{3}\cdot 3H_{2}O$$ (1)

$$3CaO\cdot 3{Al}_{2}O_{3}\cdot {CaSO}_{4}\;+\;6Ca{\left(OH\right)}_{2}\;+\;8Ca{SO}_{4}+\;90H_{2}O\to 3(3CaO\cdot {Al}_{2}O_{3}\cdot 3Ca{SO}_{4}\cdot 32H_{2}O)$$ (2)

**Fig 11:**
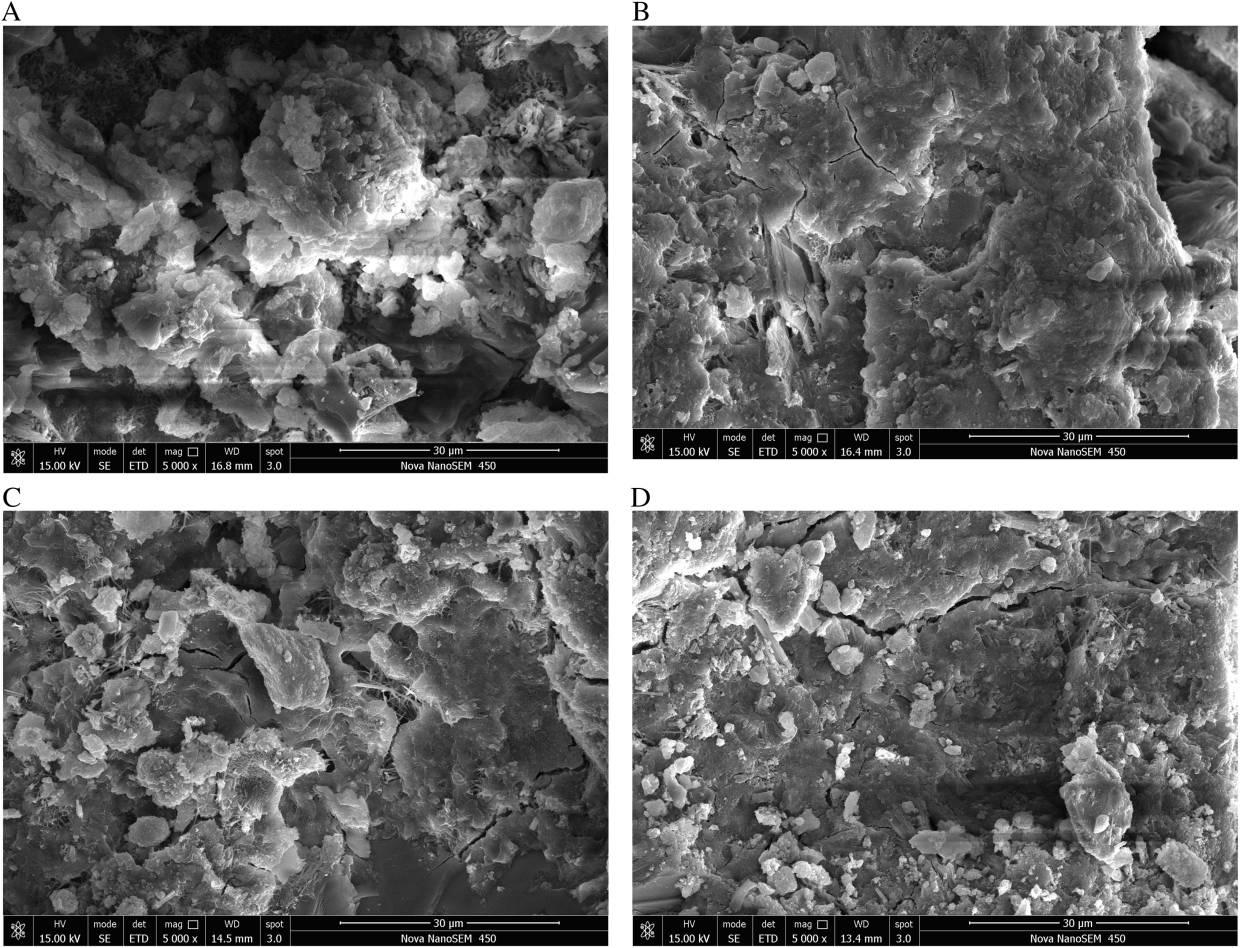
SEM images of slurry consolidation body with different expansion agent content at 5000 times. (a) Pure cement (b) 5% of the expansion agent (c) 10% of the expansion agent (d) 15% of the expansion agent.

**Fig 12:**
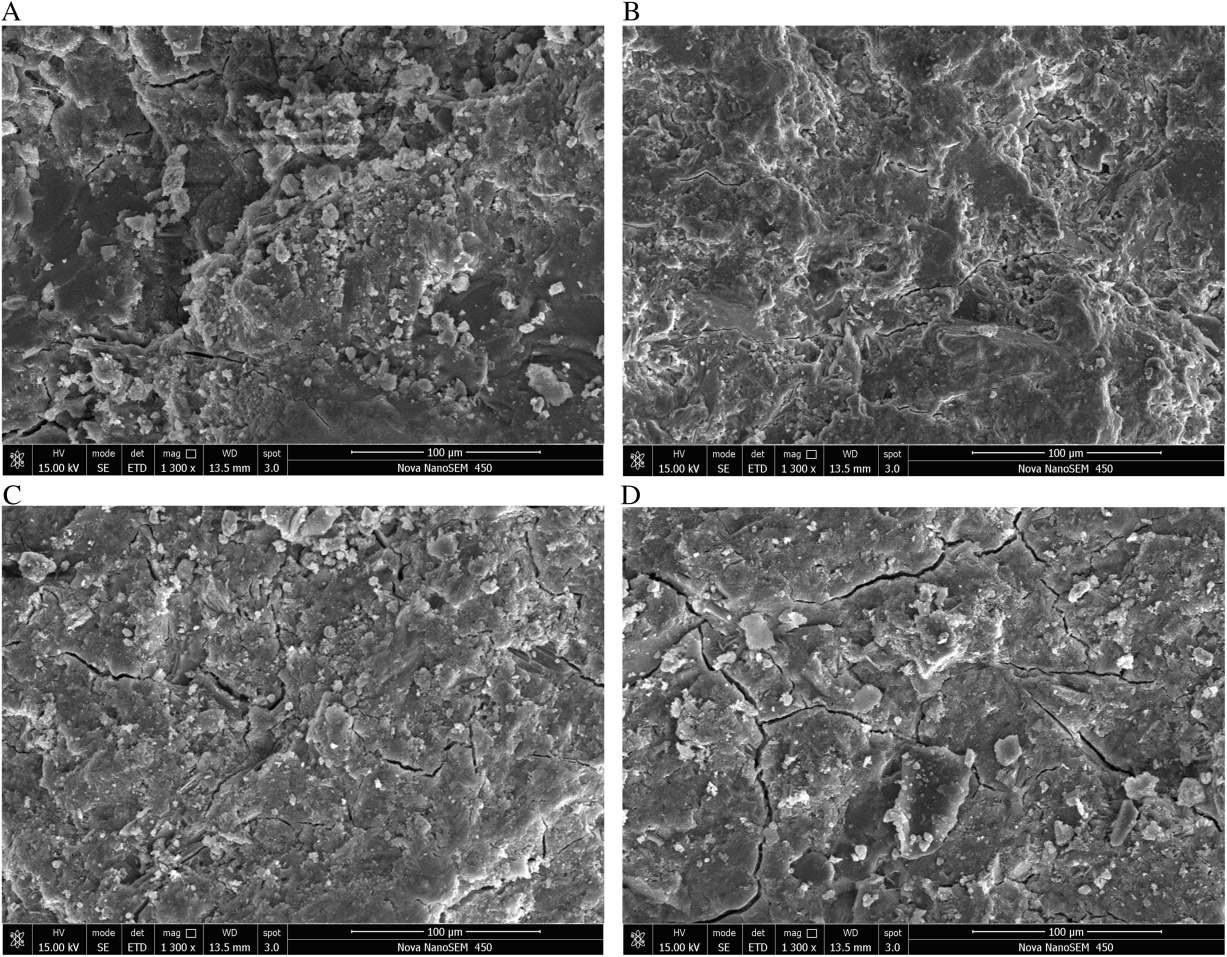
SEM images of slurry consolidation body with different expansion agent content in 1300 times. (a) Pure cement (b) 5% of the expansion agent (c) 10% of the expansion agent (d) 15% of the expansion agent.

The examination of microscopic tests primarily focuses on microstructure and phase composition. Utilize the 28-day sample to examine the microstructure and mechanism of the expansion agent for cement expansion. Samples of grouting-coal consolidation utilizing various expansion agents and grouting ratio materials were examined at magnifications of 1300 and 5000 times using scanning electron microscopy, as illustrated in Figs 11 and 12.

The pure cement generates a substantial quantity of cylindrical C-S-H gel material during hydration, hence imparting a degree of strength to the cement. Upon the addition of a specific quantity of expansion agent, calcium will be produced. The calcium content will rise with the augmentation of the expansion agent, resulting in a denser consolidated body. The resultant microcracks proliferate with the augmentation of the expansion agent. As the micro fracture expands to a specific threshold, it will result in crack propagation, thereby compromising the integrity of the consolidated structure and diminishing the strength. Consequently, when the quantity of the expansion agent rises, the strength of the grouting material initially increases and subsequently diminishes. The quantity of the expansion agent must be moderate, yielding a consolidated body strength of 28.53 MPa at 10% and 11.16 MPa at 15%.

**Fig 13:**
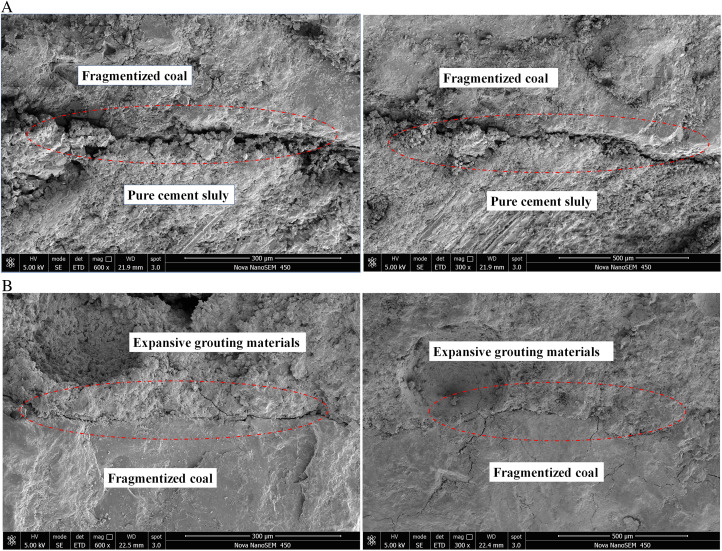
Microstructure of grouting reinforcement. (a) Pure cement reinforcement (b) expansion grouting materials reinforcement.

The grouting-coal solidity of several grouting materials were enlarged 300 and 600 times in SEM for microscopic imaging. The microstructure of the expansion grouting materials and the pure cement material utilized for reinforcing the coal body is studied, as illustrated in Fig 13.

Through microstructure analysis of grouting reinforcement with different grouting materials in Fig 3. Compared with traditional pure cement grouting materials, expansive grouting materials exhibit tighter bonding with the coal mass and smaller interfacial gaps. The coal body, augmented by the expansion grouting materials, exhibits a denser structure, and achieves effective adhesion. The interface area between the grouting material and the coal body is extensive, resulting in a robust adhesion force. expansion grouting materials may efficiently occupy the voids in fractured coal bodies, thereby reducing the porosity in the consolidated coal mass. When the grouting-coal is exposed to pressure and shear stress, friction occurs between the expansion grouting materials and the coal body. The expansion grouting materials enhances the compressive and shear strength of the coal body, demonstrating its effective reinforcing of the fractured surrounding rock.

### 3.4 Field test and analysis

#### 3.4.1 Initial support and crushing situation of the roadway.

**Fig 14:**
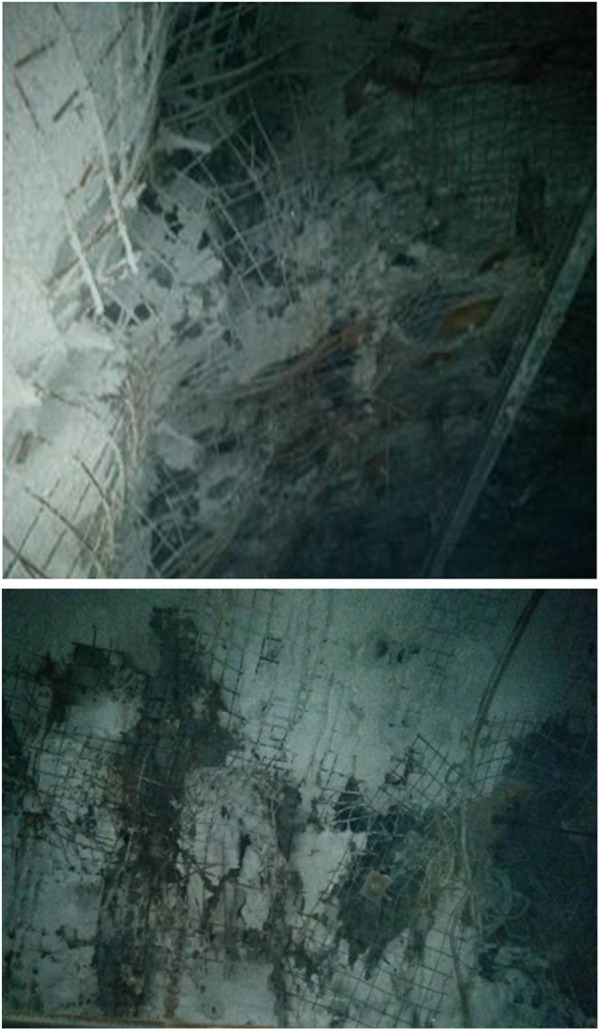
Roadway crack.

During the excavation of the 1304 tracking tunnel at Coal Mine, significant deformation was observed at the intersection of the tunnel and -808m ingate, as illustrated in Fig 14. The surrounding rock is fractured, and the loose ring surpasses the bolt’s anchorage capacity. The recently formulated grouting substance is employed to strengthen the roadway.

#### 3.4.2 Design of supplementary grouting support for roadway.

**Fig 15:**
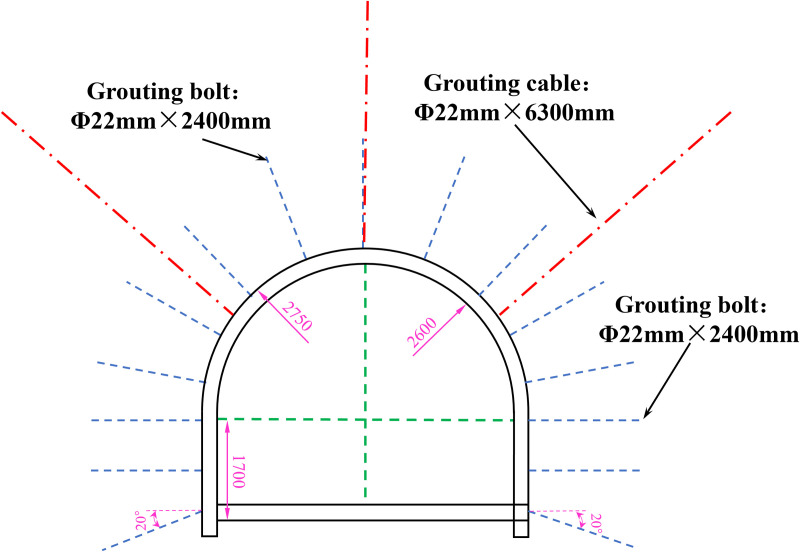
Layout of grouting borehole.

The reinforcement method of “surrounding rock grouting bolt + grouting cable” is implemented. The size of the hollow grouting bolt utilized is Ф 22 mm × 2400 mm. The grouting cable is Ф 22 mm × 6300 mm. The grouting material contained 10% expansion agent, 5% early strength agent, and 1.2% water-reducing agent, and 425# cement. The water-to-ash ratio was 0.6, and the grouting pressure was maintained at 2–3 MPa. Fig 15 illustrates the configuration of the grouting cables and grouting bolts. The spacing of the grouting cables is 1600 mm × 1600 mm, the spacing of the grouting bolts is 800 mm × 800 mm. The diameter of the drilling hole is 32 mm.

#### 3.4.3 Monitoring and analysis.

**Fig 16:**
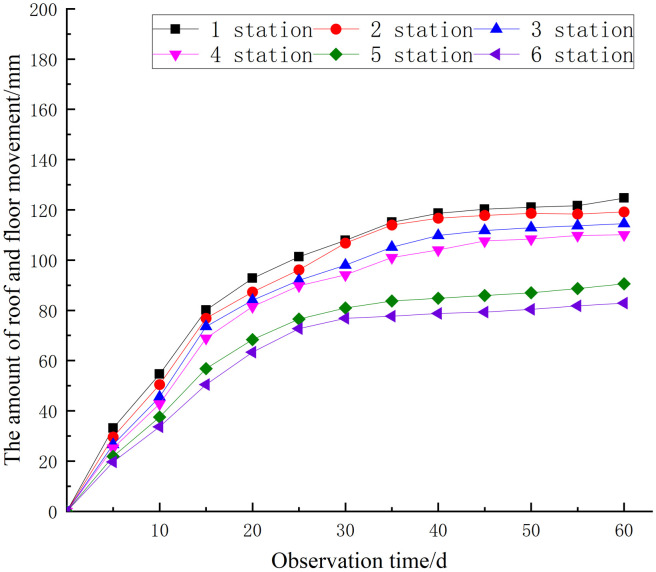
Roof-floor displacement.

**Fig 17:**
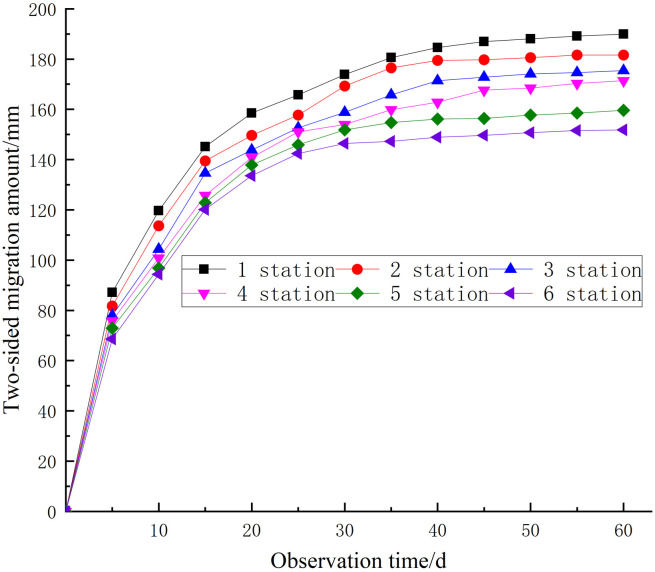
Sidewall displacement.

One measuring station is positioned every 50 meters along the street, resulting in a total of six measuring stations. The roadway’s surface displacement was quantified over a period of 60 days utilizing the cross-point method. The monitoring outcomes are presented in Table 4. The temporal change pattern of nearby rock deformation is illustrated in Figs 16 and 17.

**Table 4:** Roadway deformation.

Station	Roof-floor		Sidewalls	
	Displacement/mm	Ratio/mm/d	Displacement/mm	Ratio/mm/d
1	125	2.08	190	3.17
2	119	1.98	182	3.03
3	115	1.92	176	2.93
4	110	1.83	172	2.86
5	91	1.52	160	2.67
6	83	1.38	152	2.53

The roof-floor deformation averages 2.08 mm/d and peaks at 125 mm. The two sidewalls deform 190 mm maximum and 3.17 mm/ d average. Between days 6 and 15, the deformation rate diminished in comparison to days 0–5. The deformation rate slowed from 16 to 40 days, and the displacement curve stabilized after 40 days. Rock grouting reinforcement and support have helped, and the outcome is good.

## 4 Discussions

### 4.1 Compaction effect on backfilling

**Fig 18:**
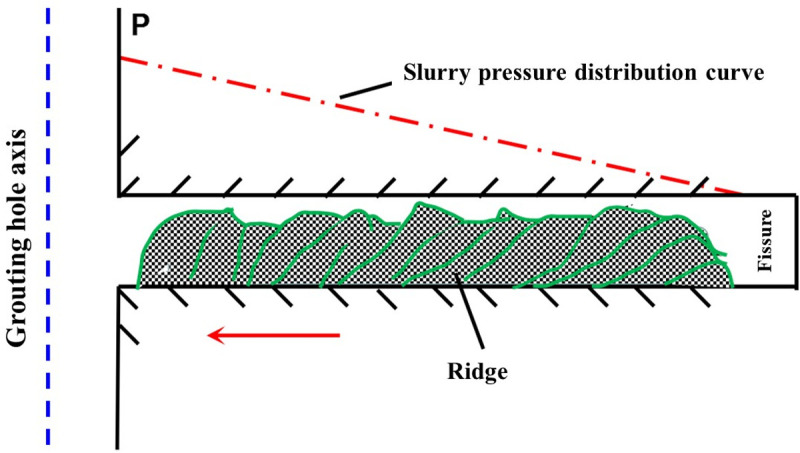
Filling mechanism of fracture expansion grouting materials in broken surrounding rock.

The filling mechanism of the expansion grouting materials in fractured surrounding rock is illustrated in Fig 18. Initially, the pressure and flow rate of the slurry progressively diminish along the crack’s diffusion direction under the grouting pressure. Upon attaining the maximum distance within the fissure, the slurry particles are segregated and commence settling. A cumulative structure progressively develops from the interior towards the exterior in the cleft. Subsequently, the width of the cumulative structure expands. Ultimately, when the grouting pressure reaches a sufficient level, the fissures are effectively filled with the slurry. The expansion grouting materials exerts pressure on the surrounding rock because to its inherent expansion force. In comparison to pure cement slurry, the expansion grouting materials increases the density of the fractured surrounding rock and the solid, hence enhancing the restoration of triaxial stress.

### 4.2 Improving the strength of surrounding rock

**Fig 19:**
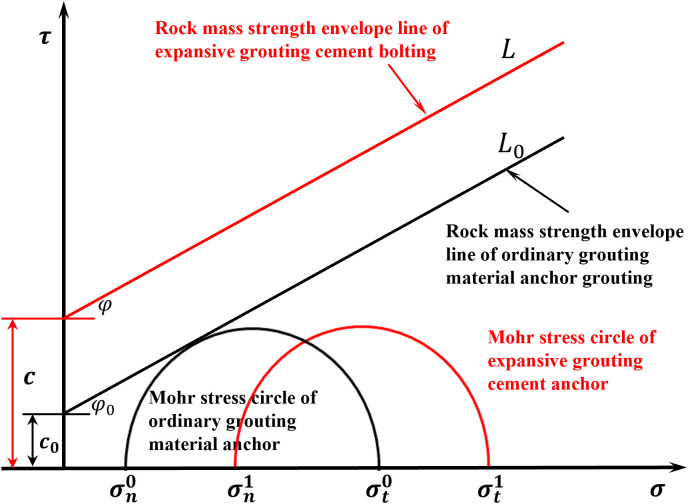
Strength curve before and after grouting reinforcement.

The expansion grouting materials exhibits certain expansion properties post-consolidation. Following grouting and strengthening, the cohesiveness and internal friction angle of the coal rock mass are enhanced. The compaction of the coal or rock mass, enhanced by expansion grouting, increases the total strength of the surrounding rock. Expansion grouting enhances the strength envelope of the surrounding rock. The expansion grouting materials possesses certain expansion properties, hence generating support for the fractured surrounding rock. Following the application of expansion grouting materials, both the maximum and minimum principal stresses within the rock mass will experience an increase to a certain degree. Consequently, the molar stress circle anchored with the expansion grouting materials is displaced to the right. Fig 19 illustrates that the distance between the rock mass strength envelope and the mole grouting circle exceeds that of conventional grouting materials. Concurrently, the enhancement of mechanical parameters, including the bonding strength of the fractured rock mass, the internal friction angle, and the resistance among the internal rock mass, is more pronounced. In comparison to conventional grouting materials, expansion grouting materials more effectively enhances the strength and load-bearing capability of surrounding rock.

### 4.3 Reducing the effect of surrounding rock loose circle

The expansion grouting materials can infiltrate deeper fissures in the fractured surrounding rock due to the influence of grouting pressure and expansion force. Upon the condensation of the slurry, a network structure emerges, sustaining the fractured surrounding rock. Expansion grouting enhances the bonding range of loose rings and reinstates it’s bearing capacity. The expansion grouting enhances the subsequent expansion of the adjacent rock loose ring. Consequently, in comparison to conventional grouting materials, the expansion grouting materials is superior in minimizing the extent of the surrounding rock’s loose ring and reinstating the load-bearing capacity.

## 5 Conclusions

This manuscript employs cement, expansion agents, early-strength agents, and water-reducing agents to develop a grouting material with specific expansion properties and strength, as demonstrated through mechanical property tests and microstructural analysis. The material effectively manages roadway deformation and restores strength. The research findings are essential for reinforcing tunnels in fractured surrounding rock and ensuring safe coal mining operations. The primary research outcomes are as follows:

(1) The expansion grouting materials fortifies the fractured surrounding rock, efficiently filling the fissures and creating a compaction effect, so enhancing the stress condition of the surrounding rock’s loose ring, increasing its strength, and minimizing the extent of damage.
(2) A novel expansion grouting materials was developed through orthogonal testing. The raw components include 425# cement, UEA expansion agent, chlorine salt early strength agent, and polycarboxylic acid efficient water-reducing agent. The optimal proportions are 10% expansion agent, 5% early strength agent, and 1.2% water-reducing agent.
(3) The recovery strength coefficient of the 7-day expansion grouting materials is 0.82, in comparison to an additional 0.18 of pure cement. The recovery strength coefficient of the reinforced fractured rock mass 7d is 0.25, exceeding that of pure cement by 0.1. The 28-day recovery strength coefficient is 1.40, exceeding that of pure cement by 0.27. The recovery strength coefficient of reinforced broken rock mass at 28 days is 0.48, exceeding that of pure cement by 0.15.
(4) The field application results indicate that the roof’s maximum displacement is 125 mm, while the sidewall exhibit a maximum deformation of 190 mm. The grouting cables and grouting bolts are firmly secured. The expansion grouting materials effectively mitigates roadway crushing and stabilizes adjacent rock.
